# One Lung Soldier: A Ventilation Conundrum in a Postpneumonectomy Syndrome Complicated by Acute Respiratory Syndrome

**DOI:** 10.1155/2020/5476794

**Published:** 2020-03-11

**Authors:** Jaskaran K. Purewal, N. F. N. Sakul, Nikhita R. Balabbigari, Alberto Nenninger, Nisha Kotecha

**Affiliations:** ^1^Department of Medicine, Overlook Medical Center, Summit, NJ 07901, USA; ^2^Department of Pulmonary Critical Care, Overlook Medical Center, Summit, NJ 07901, USA

## Abstract

Postpneumonectomy syndrome involves mediastinal shift causing dynamic airway obstruction via compression of the main bronchus and distal trachea. A few case reports describe the development of ARDS in patients with postpneumonectomy syndrome. Reeb et al. (2017) describe the mortality of postpneumonectomy ARDS anywhere from 33% to 88%. One may encounter difficulty in intubation and ventilation as parameters based on ideal body weight may not apply. Prone positioning ventilation and ECMO have been successfully used in isolated cases. We present such a case and highlight challenges in management. A 70-year-old male Vietnam veteran with remote history of right pneumonectomy thirty years prior presented with fever, cough, and dyspnea. Physical exam was significant for T 36.3°C, BP 162/73, heart rate 145 BPM, RR 22 breaths/minute, ht. 1.72 m, and wt. 78 kg, with transmitted right lung sounds and rhonchi on the left. Labs showed WBC 23.92/nL and procalcitonin 0.84 ng/mL. CXR showed left infiltrate and opacification of right hemithorax with right mediastinal shift. EKG showed atrial fibrillation. He was started on broad spectrum antibiotics for pneumonia, but deteriorated, and was intubated for respiratory distress from ARDS. Vasopressors were initiated for shock. Given the history of pneumonectomy, he was initially ventilated with lower tidal volumes (320 mL). However, incremental changes were made to tidal volumes, and ETT was repositioned several times for hypoxia. Epoprostenol and cisatracurium were also initiated. Positional changes would lead to sudden desaturation; hence, prone positioning ventilation was not done. He was not considered for ECMO due to his pneumonectomy status. Unfortunately, his condition worsened progressively and he expired. The guidelines for ARDS are well established. However, postpneumonectomy patients are unique as seen in our patient. It is unclear whether an endobronchial tube advanced into the left bronchus could have helped difficult airway management resulting from suspected postpneumonectomy syndrome as suggested by CXR. Higher tidal volumes were also unsuccessful in alleviating hypoxia and led to persistently elevated plateau pressures and driving pressures as high as 23, which was inconsistent with our goal of lung protective ventilation. Few case reports describe the successful use of prone positioning ventilation or ECMO in postpneumonectomy patients with ARDS. Although not well studied, low tidal volumes supported with ECMO may have been a favorable strategy for our patient.

## 1. Introduction

The postpneumonectomy state is a unique entity with associated anatomical and physiological challenges. One such challenge is the postpneumonectomy syndrome, more commonly seen in patients with right pneumonectomies, which involves dynamic airway obstruction resulting from compression of the main bronchus and distal trachea by the vertebral column or aorta due to the rotation and right mediastinal shift. These patients pose particularly difficult challenges in ventilation due to the increased respiratory dead space and decreased venous return and are prone to shunting and development of pulmonary edema [1]. The management of ARDS in these patients is not well established but usually is conservative and often involves mechanical ventilation once it becomes necessary. Due to the unique anatomy and physiology, one may encounter difficulty in endotracheal tube positioning and selecting ventilation parameters. Studies regarding ventilator management in ARDS excluded patients with pneumonectomy. The usual lung protective ventilation parameters dependent on ideal body weight may not apply to patients with a single lung. But at the same time, the attempt to lower the tidal volume further may be impossible due to difficulty in oxygenation of such patients. Prone positioning ventilation and extracorporeal membrane oxygenation (ECMO) have been successfully used in isolated similar cases but have been generally reserved for postsurgical pneumonectomy patients who develop ARDS. In a small retrospective study by Reeb et al., the use of VV-ECMO in postpneumonectomy ARDS patients was studied and subsequently showed improved hospital survival [2]. We present a patient with remote history of pneumonectomy now admitted with ARDS and highlight unique challenges associated with its management.

## 2. Case Presentation

**Figure 1:**
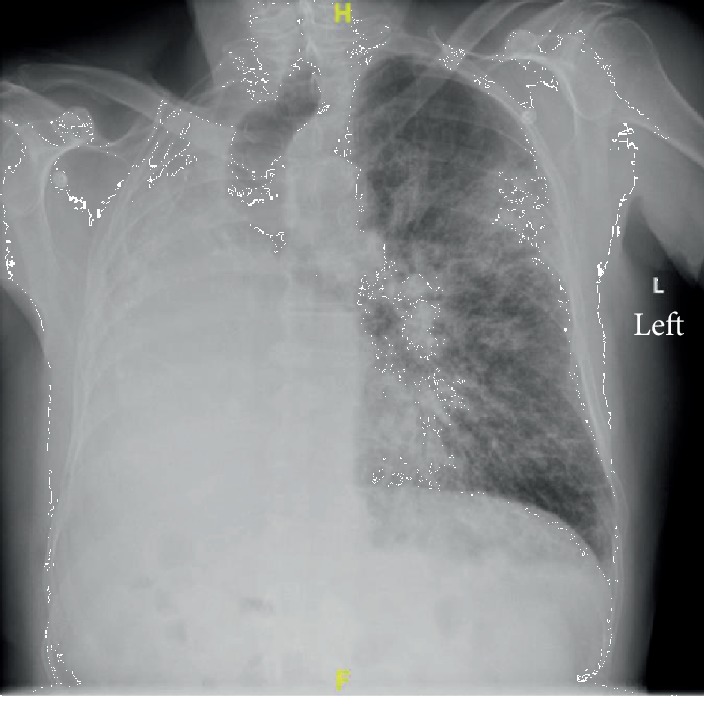
Initial chest X-ray showing complete opacification of the right hemithorax from pneumonectomy. Right mediastinal shift present. Diffuse infiltrates noted in the left lung.

**Figure 2:**
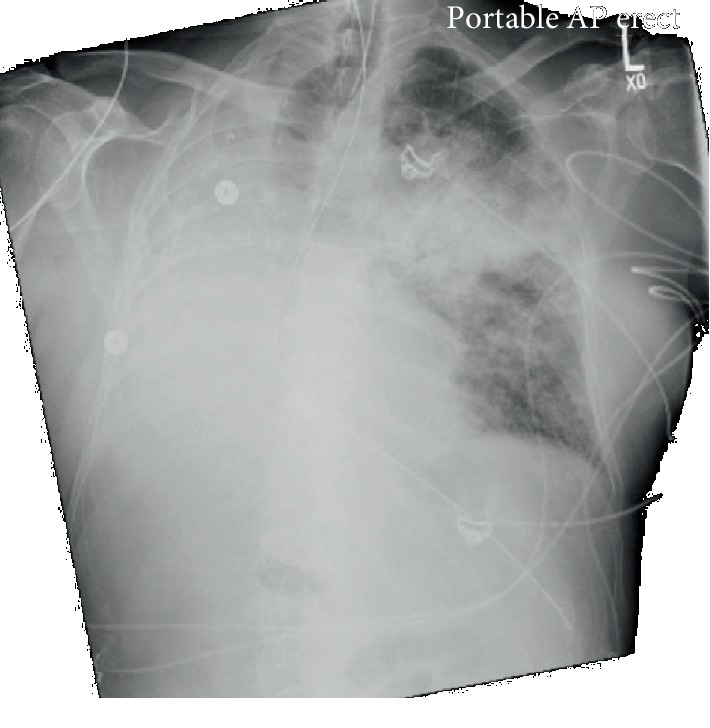
Repeat chest X-ray done one day after admission. Patient noted to have worsening hypoxia and increased work of breathing and was subsequently intubated. Chest X-ray significant for worsening of the left-sided superior perihilar infiltrate.

**Figure 3:**
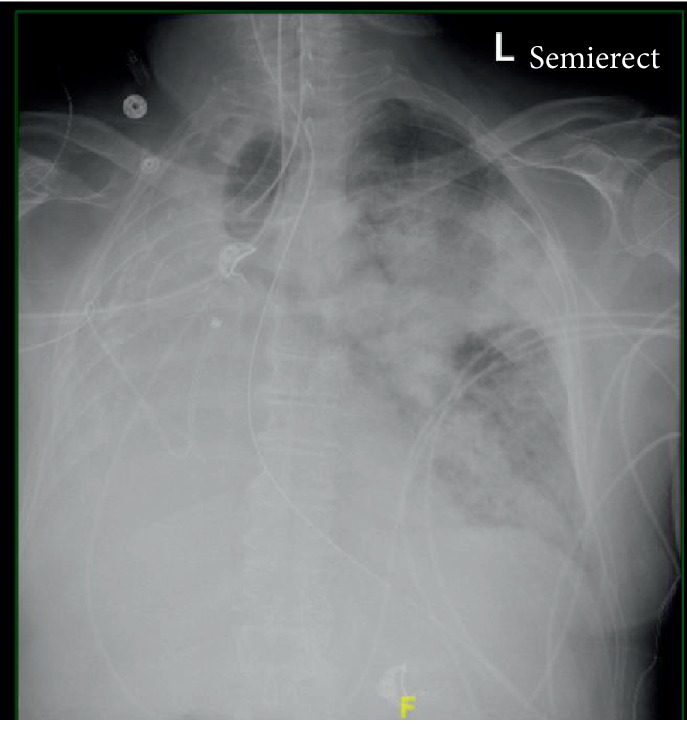
Chest X-ray done after advancement of the endotracheal tube, shown in the image to be projected toward the right side of the trachea. Persistent airspace disease noted in the left upper lobe.

**Figure 4:**
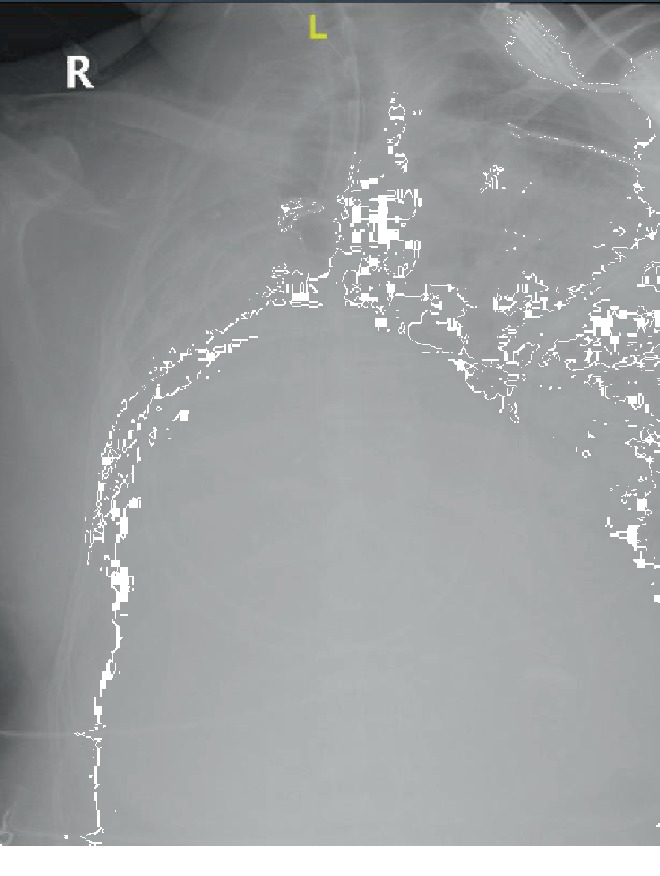
Chest X-ray showing significant worsening of the L-sided infiltrate, leading to complete opacification of the left lung.

A 70-year-old male with hypertension and remote history of carcinoid tumor requiring right pneumonectomy thirty years prior presented with one week of generalized malaise, fever, nonproductive cough, and worsening dyspnea on exertion. He was in his usual state of health until one week prior to his presentation; overall, he was very healthy with good functional status with slightly decreased stamina. Initial physical exam was significant for temperature 36.3°C, BP 162/73, irregular heart rate 145 beats/minute, respiratory rate 22 breaths/minute, height 1.72 m, and weight 78 kg. He was a thin male with transmitted lung sounds on the right and significant rhonchi on the left. The remainder of the physical exam was otherwise within normal limits. Initial lab results showed leukocytosis (WBC 23.92/nL), positive procalcitonin (0.84 ng/mL), negative Legionella and S. pneumoniae urine antigen, negative respiratory viral panel, and mild respiratory alkalosis on ABG (7.49/39/69/24.6). EKG showed atrial fibrillation with rapid ventricular response. Initial CXR showed left-sided infiltrate with pleural effusion as well as complete opacification of the right hemithorax with right mediastinal shift (Figure 1). Overall, there was concern for severe sepsis due to multilobar community-acquired pneumonia. He was subsequently started on ceftriaxone and azithromycin and placed on high-flow nasal cannula. He was also started on a continuous infusion of diltiazem and received full-dose enoxaparin for anticoagulation in the setting of uncontrolled atrial fibrillation. He quickly converted to normal sinus rhythm within a few hours and was started on oral diltiazem. However, within four hours of hospital admission, his respiratory status quickly deteriorated and a rapid response was called for hypoxia and increased work of breathing. Oxygen requirements on high-flow nasal cannula had increased significantly, and while he was able to speak in full sentences, he was clearly anxious, air hungry, and using accessory muscles to breathe. ECHO showed normal left ventricular function with an ejection fraction of 55% and a sclerotic aortic valve without stenosis but was not significant for any cardiac shunt, such as patent foramen ovale. Repeat ABG showed worsening hypoxia with a PaO_2_ : FiO_2_ ratio of 71 mmHg. Repeat CXR showed worsening of the left-sided infiltrate (Figure 2). Further imaging with CT chest or even a bronchoscopy was needed to further qualify the worsening infiltrate as well as possible obstruction from postpneumonectomy syndrome; however, the patient was never hemodynamically stable for this. He required transfer to the intensive care unit and intubation for significant respiratory distress, with concern for developing ARDS and septic shock. Antibiotic coverage was broadened to vancomycin, piperacillin-tazobactam, and azithromycin; stress dose steroids and vasopressors were also initiated. Given the history of pneumonectomy, he was initially ventilated with low tidal volumes (320 mL). While the goal was to maintain lung protective ventilation, incremental changes were made to his tidal volume to maintain oxygenation but were not effective. Throughout his ICU course, oxygenation and ventilation proved to be difficult. In addition, his endotracheal tube was repositioned several times leading to temporary improvements in oxygenation (Figure 3). Epoprostenol and neuromuscular blockade were initiated for oxygenation and ventilator synchrony; however, they were only beneficial temporarily. Furthermore, peak and plateau pressures were consistently elevated, suggestive of an obstructive component, possibly from worsening edema from ARDS exacerbating his underlying postpneumonectomy state. While proning ventilation was considered, it was not done as even minimal changes in his position lead to sudden hemodynamic instability with desaturation and tachycardia. Furthermore, due to his remote history of pneumonectomy and clinical severity, he was not considered a good candidate for ECMO. Unfortunately, due to his progressively worsening clinical condition, which was also verified by the worsening chest X-ray (Figure 4), he was transitioned to comfort care measures and expired.

## 3. Discussion

ARDS is a well-recognized clinical entity in an ICU setting, and its management involves treatment of the underlying etiology as well as ventilation management. Several guidelines and studies have been conducted to direct treatment and escalation of care to prone ventilation and ECMO. However, postpneumonectomy patients have particular anatomical and physiological changes that pose challenges in their management of ARDS as seen in our patient.

Pneumonectomies are indicated for numerous reasons including removal of malignancy or in a trauma setting. While uncommon, postpneumonectomy syndrome can occur within weeks or even years after removal of the lung. It is more commonly seen after right pneumonectomy, possibly due to the more severe anatomical changes. The postpneumonectomy syndrome results in airway obstruction due to rotation and mediastinal shift towards the pneumonectomy site. This results in eventual herniation and overinflation of the remaining lung and leads to central airway compression [3]. Patients will often present with stridor, worsening dyspnea, or recurrent pulmonary infections as a result. This was clearly seen in our patient. Imaging findings clearly described with relation to postpneumonectomy syndrome were clearly also illustrated in our patient's initial admitting chest X-ray (Figure 1). While he was asymptomatic from the clear anatomical changes noted on his imaging, infection and subsequent edema may have exacerbated his underlying postpneumonectomy state. In a study done in 2008, surgical correction of the syndrome with prostheses led to improved flow rates, decreased FVC, and correction of the hyperinflation, as well as an improved FEV1/FVC ratio. However, more than the changes noted on PFTs, patients experienced significant improvement in their symptoms [3]. It is clear that this syndrome causes airway compression, and thus, airway management may be difficult. Besides the difficulty in intubation from mechanical obstruction, the associated hypercapnia, hypoxia, increased respiratory dead space, and overall decreased venous return can contribute to ventilation problems, as was seen in our patient [1]. Initial airway management was difficult due to poor placement of the ETT (Figures 2 and 3). While a CT chest would have been ideal to visualize the extent this played in his ventilation problems, it was not feasible. A similar case was described in 2015, in which a patient underwent a left-sided pneumonectomy for a persistent mycobacterial infection and three months later became symptomatic. CT imaging in this patient showed significant obstruction of the bronchus intermedius and right lower bronchus. The case report also describes difficulty in oxygenating the patient, and eventually, the patient's endotracheal tube was then temporarily replaced with an endobronchial tube until surgery was viable. The authors additionally describe considering the use of an endobronchial stent but mention uncertainty in its efficacy or long-term risks [4]. In the anesthesia literature, mediastinal masses often create airway obstruction, which are managed with the use of endobronchial tubes [5]. Whether a single lumen endobronchial tube advanced up to the left bronchus could have been used to decompress the airway and overcome this obstruction to provide better ventilation remains unclear. Another potential concern was a possible platypnea-orthodeoxia syndrome caused by interatrial shunting after pneumonectomy [6]. However, this was highly unlikely as no intracardiac shunt was noted on ECHO, and furthermore, our patient was not symptomatic from this condition prior to presentation, even in the setting of his pneumonectomy done thirty years ago.

Since the PROSEVA trial in 2013, early initiation of proning in patients with severe ARDS had an associated mortality benefit [7]. However, there is limited literature regarding postpneumonectomy patients affected with ARDS and the subsequent effect of proning. While prone ventilation was not an option for our patient due to hemodynamic instability, case reports have demonstrated significant improvement in similar patients [8, 9]. Furthermore, it is uncertain whether typical ventilation strategies used in ARDS management may be as effective due to a combination of the underlying pathophysiology of postpneumonectomy syndrome and ARDS. Initial ventilator settings were to maintain low tidal volumes and were adjusted based on the patient's pneumonectomy status; we still had difficulty maintaining adequate oxygenation despite adjustments and increases to the tidal volume. These increments in tidal volume led to persistently elevated plateau pressures > 30 and driving pressures as high as 23, which oppose the goal of lung protective ventilation. Low tidal volumes when supported with ECMO may have been a favorable strategy for our patient and allowed us to ventilate with a lower plateau and driving pressures. A small study done by Reeb et al. showed that the use of VV-ECMO in postpneumonectomy ARDS patients decreased the mortality from 80 to 50%. ECMO had allowed for greater lung protective strategies, decreased tidal volumes, adequate gas exchange, and overall better recovery of lung tissue [2]. ECMO was not considered in our patient but may have been beneficial and may have allowed for the use of improved lung protective strategies. While it is difficult in this particular set of patients, greater study is needed regarding ARDS management in postpneumonectomy patients.

## 4. Conclusion

Few case reports describe improved mortality with prone ventilation as well as ECMO in postpneumonectomy ARDS, which in hindsight may have been beneficial for our patient given the difficulties we had in managing our patient's ventilation. While this has not been well studied due to the unique nature of these disease states, it is important to keep in mind as a possibility for management in postpneumonectomy patients. Our case highlights how better guidelines with regard to treatment concerning single lung ventilation and a possible postpneumonectomy syndrome may have been life changing in his scenario.

## Abbreviations

ECMO: Extracorporeal membrane oxygenation
ARDS: Acute respiratory distress syndrome
CXR: Chest X-ray
EKG: Electrocardiogram
ABG: Arterial blood gas
WBC: White blood cell
FEV1: Forced expiratory volume
FVC: Forced vital capacity
PFTs: Pulmonary function tests
ETT: Endotracheal tube
VV-ECMO: Venovenous extracorporeal membrane oxygenation.

## Additional Points

*Key Points*. Postpneumonectomy syndrome is a rare entity that leads to mechanical airway obstruction due to mediastinal shift, which can contribute to difficult airway management. The use of prone ventilation and ECMO have been used in isolated cases of postpneumonectomy ARDS, but their utility and subsequent effects are not well understood. Further study is needed on ventilation in patients with pneumonectomies.
